# Proteomic Analysis of Differentially-Expressed Proteins in the Liver of Streptozotocin-Induced Diabetic Rats Treated with *Parkia biglobosa* Protein Isolate

**DOI:** 10.3390/molecules23020156

**Published:** 2018-01-24

**Authors:** Bolajoko Idiat Ogunyinka, Babatunji Emmanuel Oyinloye, Foluso Oluwagbemiga Osunsanmi, Andrew Rowland Opoku, Abidemi Paul Kappo

**Affiliations:** 1 Biotechnology and Structural Biology (BSB) Group, Department of Biochemistry and Microbiology, University of Zululand, KwaDlangezwa 3886, South Africa; bolajokotimi@gmail.com (B.I.O.); tunji4reele@yahoo.com (B.E.O.); alafin21@yahoo.com (F.O.O.); opokua@unizulu.ac.za (A.R.O.); 2 Department of Biochemistry, College of Sciences, Afe Babalola University, PMB 5454, Ado-Ekiti 360001, Nigeria

**Keywords:** MALDI-TOF-MS, nLC-MS, *Parkia biglobosa*, proteomics, rat liver, STZ-diabetes

## Abstract

Protein isolate from *Parkia biglobosa* seeds is believed to possess excellent anti-diabetic properties. The purpose of this study was to identify differentially expressed proteins in liver of streptozotocin-induced diabetic rats treated with *Parkia biglobosa* seeds protein isolate (PBPi). In this study, total proteins extracted from rat liver were separated on one-dimensional SDS polyacrylamide gel (1D SDS-PAGE) and stained with Coomassie brilliant blue (CBB) to visualize protein bands. We observed that protein bands in the region of 10–15 kDa were altered by the different treatments; these bands were selected and excised for in-gel digestion and peptide extraction followed by nLC-MS, MALDI-TOF MS, and LIFT MS/MS. A database search with the Mascot algorithm positively identified four differentially expressed proteins. These proteins are known to be responsible for diverse biological functions within various organs and tissues. The present result gives insight and understanding into possible molecular mechanisms by which streptozotocin causes various alterations in proteins found in the liver of diabetic rats and the possible modulatory role of PBPi in the management of streptozotocin-induced diabetes.

## 1. Introduction

Multiple-organ failure has emerged as one of the most significant cause of death associated with diabetes mellitus in both developing and developed countries of the world [1]. Diabetes can be classified into two broad categories: type 1 diabetes (insulin dependent diabetes mellitus) and type 2 diabetes (non-insulin dependent diabetes mellitus) [2]. Studies have shown that in 2010, about 280 million individuals had diabetes with type 2 comprising of about 90% of the total cases worldwide; while it has been projected that by 2030, the number of individuals suffering from diabetes would have increased to about 430 million, due to the recent increase in prevalence of this disease globally [3,4]. 

Diabetes mellitus is an endocrine metabolic disorder characterized by abnormal increase in the blood sugar level (hyperglycaemia) as well as alteration in lipids, carbohydrates, and protein metabolism, which is linked to low level of insulin production or insensitivity of target organs to insulin [5,6]. Sustained hyperglycemia can lead to disturbances in the cell structure and functions of organs [7]. It triggers the activation of polyol pathway, enhances sorbitol and fructose accumulation, increases intracellular formation of advanced glycation end products, activates protein kinase C and nuclear factor-kappaB (NF-κB), and increases hexosamine pathway flux. This cascade of events leads to oxidative stress as a result of overproduction of superoxide radicals by the mitochondrial electron transport chain [8,9].

Oxidative stress in the biological system is a state of imbalance that occurs when the level of free radicals (notably, reactive oxygen species) production exceeds cellular antioxidants capacity as a result of various metabolic abnormalities within the body [2,10]. Hyperglycemia-induced oxidative stress accounts for the majority of diabetic complications [1]. From time immemorial, medicinal plants have been used by traditional healers who believe in their potency for maintaining good health in the treatment of various ailments including diabetes and its associated complications [7,11]. Medicinal plants have constantly been an excellent source of drugs and several of the currently obtainable drugs have been derived directly or indirectly from plants [12]. *Parkia biglobosa* is one of them. It is commonly used in Nigeria and many other West African countries as a spice for the flavoring of foods [2].

It was recently reported that *P. biglobosa* (aqueous and methanolic) extracts possess some active phytochemicals which are responsible for its nutritional and pharmacological effects such as hepatoprotective, hypoglycemic, hypolipidemic, antimicrobial, and anti-inflammatory activities in experimental animal models [2,13,14]. In addition, PBPi have been identified and believed to possess various promising insulin-like proteins with insulin-releasing activity in a sub-acute toxicity study conducted by oral administration of PBPi at daily doses of 200 or 400 mg/kg body weight for 28 days [15]. The liver acts as a storehouse for glycogen, which is required for maintaining the level of blood sugar and the needs of the body. More so, the liver plays an important role as it acts to equilibrate the uptake and storage of glucose via glycogenesis and regulates the release of glucose by activating glycogenolysis and gluconeogenesis [16]. To understand the underlying molecular and cellular mechanisms of diabetes and systemic cellular alterations in health and diseases, it is important to evaluate the level of alterations in the composition of proteins in cell and tissues [7,17].

Differential protein expression analysis is the most recent and powerful experimental approach that enables a systematic and comparative analysis of proteomic changes [7,17]. This approach uses a combination of two-dimensional gel electrophoresis (2-DE); image analysis; matrix-assisted laser desorption/ionization-time of flight (MALDI-TOF) mass spectrometry (MS); and bioinformatics analyses to comprehensively resolve, identify, and characterize proteins in the cells, tissues, and animal models [18]. Karthik and co-workers [7] documented that the term ‘‘differentially expressed protein’’ is used to indicate that a protein, which plays a precise biological role, was significantly decreased or increased in concentration when compared with normal control. Differential expression can be an outcome of disease-related alterations in transcription, protein synthesis, transport, degradation, and/or covalent modification [17].

This proteomic approach has been employed to ‘fish’ out proteins that are responsible for, or that are related to, abnormal functions in various cells and tissues [17]. In this proteomic study, we analyze the proteomic changes in the liver of STZ-induced diabetic rats and the possible effect of *Parkia biglobosa* seeds protein isolate (PBPi) treatment at varying doses.

## 2. Results

**Figure 1:**
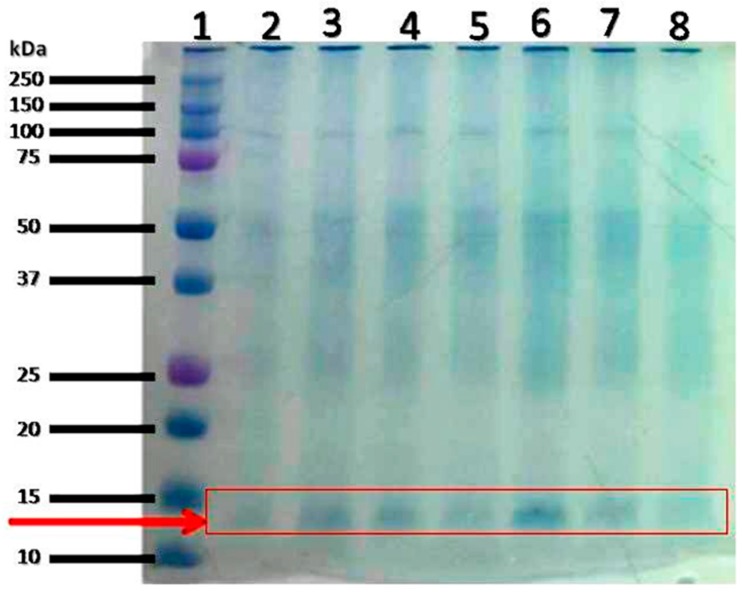
1D SDS-PAGE gel from the liver of the experimental animals. Proteins (20 μg) were separated by 1D SDS-PAGE and visualized by CBB staining. The protein profiles of four representative samples are shown in this figure. Lane 1: Molecular marker; lane 2: STZ PI 200; lane 4: STZ; lane 6: Control and lane 8: STZ PI 400.

Total protein extract from the liver of the experimental animals was separated on 1D SDS-PAGE gel. The molecular weight distribution of the total protein from the liver of the experimental animals (healthy control, untreated diabetic rats, and treated diabetic rats; 200 or 400 mg/kg bw PBPi) is shown in Figure 1. The proteins were well separated and based on the appearance on the gel; the protein bands in the region of 10–15 kDa appeared to be altered by the different treatments; these bands were selected and cut for in-gel digestion and peptide extraction followed by nLC-MS, MALDI-TOF MS, and LIFT MS/MS in order to identify the proteins.

Protein identification was carried out by database interrogation. The peptide masses of each protein sample obtained by MALDI-TOF MS and LIFT MS/MS were submitted to MASCOT and searched against the Swiss-Prot database. This resulted in the positive identification of four differentially expressed proteins as shown in Table 1 and Table 2 (candidate protein matches with a MOWSE score greater than 22 were considered as identified proteins).

## 3. Discussion and Conclusions

Diabetes mellitus is a group of metabolic irregularities characterized by hyperglycemia, emerging at an alarming rate globally with serious health complications [4,19]. Many of the current therapies for diabetes (insulin and other oral anti-diabetic agents) have some limitations and side effects, such as hypoglycemia, diarrhea, and liver and kidney failure, just to mention a few [19]. At the moment, there is considerable number of studies studying the potential benefits of plants and plant extracts in the prevention, treatment, and management of diabetes and its related complications [2,20,21]. It is recently believed that alterations at molecular level (protein structure, function, production, and interactions) play an important role in part—if not completely—in the pathogenesis of many metabolic diseases including diabetes; and that these alterations contribute significantly to the underlying mechanism of these diseases [22].

In view of the fact that diabetes is a polygenic disease with expected alterations in many proteins; proteomics is a powerful approach to study the pathophysiological mechanisms of alteration in these proteins as well as the mechanism of action of plant-derived remedies in STZ-induced diabetic rats [23,24,25]. The present study demonstrated that STZ-induced diabetes resulted in a decreased expression of most of the proteins identified in this study. Treatment with PBPi (200 or 400 mg/kg bw) substantially upregulated their expression in a dose-dependent manner. This complemented the previously identified protective role of *Parkia biglobosa* protein isolate in human health and disease. The four proteins that were focused on in this report were either upregulated by PBPi or down-regulated by STZ-treatment in this study. In Table 2, the four proteins were compared in all groups, what is presented on the table is the effect of STZ down-regulating some of the proteins while PBPi is contributing to the enhanced abundance of some of these proteins.

Thioredoxin OS = Rattus norvegicus GN = Txn PE = 1 SV = 2 (THIO_RAT) is one of the differentially expressed proteins identified in the present study. The biological system possess elaborate defense mechanisms which prevent cellular damage; this involves metabolic enzymes such as superoxide dismutase, catalase, and glutathione peroxidase for free radical detoxification as well as non-enzymatic molecules such as thioredoxin, thiols, vitamins E and C, and trace metals such as selenium which function as direct scavengers of ROS [26]. Thioredoxin alterations have been reported in many diseases, including diabetes and its related complications [27]. 

Hyperglycemia promotes the thioredoxin/Txnip interaction leading to a functional inhibition of the thioredoxin antioxidative role. This particular interaction alters the cellular redox balance and enhances intracellular oxidative stress [28]. We believe the downregulation of thioredoxin in the treated groups is as a result of the overwhelming effect of ROS generation on the endogenous thioredoxin system. Although at the stage of this report, we are not sure of the thioredoxin subtype that was expressed in this study. However, we envisage that when the current on-going study in our laboratory is completed, we may be able to distinguish and report the thioredoxin subtype that was expressed in this study. In spite of the aforementioned, we suggest that one of the mechanisms by which STZ may elicit its deleterious metabolic effect is through the down regulation of the endogenous thioredoxin activity.

Profilin-1 OS = Rattus norvegicus GN = Pfn1 PE = 1 SV = 2 (PROF1_RAT) was also identified to be differentially expressed in this study. Profilin-1 is a small ubiquitous actin-binding protein, (12 to 15 kD) widely distributed in various types of cells with highly conserved sequences [29,30]. It binds G-actin and motivates the exchange of ADP to ATP on G-actin, thus controlling the pool of ATP-actin within the cell [30]. Profilin-1 plays a significant role in endothelial dysfunction and atherosclerosis; and endothelial dysfunction has been identified and implicated as an underling factor preceding structural alterations in diabetic vascular disease, although its molecular basis is still poorly understood [31].

Added to this, upregulation of profilin-1 has been documented in the aortic endothelium of diabetic individuals and in STZ-induced diabetic animal models [32]. In this study, induction of diabetes using STZ also increased the expression of profilin-1 in the diabetic untreated group while treatment with PBPi at the tested doses decreased the expression of profilin-1. This is an indication that PBPi can prevent or delay various complications associated with diabetes.

Furthermore, mitochondrial pyruvate carrier 1 OS = Rattus norvegicus GN = Mpc1 PE = 2 SV = 1 (MPC1_RAT) was differentially expressed by the different treatments in this study. Mitochondrial pyruvate carriers (MPC) are extremely conserved proteins that are essential for pyruvate uptake at the mitochondrial inner membrane [33]. The MPC complex comprises two paralogous subunits, MPC1 and MPC2; approximately 12 kDa and 14 kDa with two and three predicted transmembrane domains, respectively [34]. Even though their existence has been known for over 40 years, it is only recently that these proteins are identified at the molecular level [35,36]. Their contribution in the control of cell fate in cancer and gluconeogenesis in type 2 diabetes models has been demonstrated in recent documented reports. Therefore, biochemical evaluations of MPC activities are foundational for understanding the role of pyruvate transport in health and disease [34].

Though little is known about how pyruvate is transported into the mitochondria in β-cells, it is well established in literature that mitochondrial metabolism of pyruvate is crucial for insulin secretion [37]. Loss of either MPC1 or MPC2 protein may result in destabilization and degradation of the other and consequently loss of the MPC complex. It was recently demonstrated that the MPC might be a point of altered metabolic regulation in disease states such as cancer, obesity, and type 2 diabetes [35]. Pyruvate is the end product of glycolysis and a fundamental metabolite linking lipid synthesis, amino acid biosynthesis, and gluconeogenesis [38]. Although, gluconeogenesis plays a vital role in blood glucose homeostasis during fasting, it has also been implicated in sustaining hyperglycemia in insulin resistance and type 2 diabetes therefore, maintaining a critical balance between energy demand and supply is essential for health [39].

Owing to the central metabolic position occupied by the MPC, alterations in MPC activities—either during post-translational modifications or protein abundance—may intensely regulate overall cellular metabolism. In this study, it is worth noting that MPC 1 was not expressed at all in the control, STZ and STZ PI 200 groups. Interestingly, treatment with PBPi 400 mg/kg bw contributed to the enhanced abundance of this protein in the STZ PI 400 group. Taking to account the MOWSE score, the number of peptides, and the sequence coverage of this protein in our diabetic animal model in this experiment, we believe the increased expression of this protein is not detrimental, as it will contribute positively to the metabolic regulation of pyruvate in this disease state. We suggest that this is one of the mechanisms by which PBPi exhibits its protective effect in the prevention and management of diabetes.

Additionally, fatty acid-binding protein, liver OS = Rattus norvegicus GN = Fabp1 PE = 1 SV = 1 (FABPL_RAT) was also found to be differentially expressed by the different treatments in this study. Fatty acid binding proteins (FABPs) are abundantly expressed as 14–15 kDa proteins. They are intracellular lipid chaperones whose biological role and mechanisms of action are still poorly understood, but they are believed to be a group of molecules that coordinate lipid responses in cells and they are also believed to be strongly connected to metabolic and inflammatory pathways [40,41]. FABPs are known to facilitate the utilization of lipids in metabolic pathways in adipocytes and other cells. It has been suggested that they play an essential role, probably a link between lipid metabolism, hormone action, and cellular functions in adipocytes and other cell types, where they contribute to systemic energy homeostasis [41].

In the present study, based on the number of peptides, sequence coverage, and MOWSE score—the results obtained demonstrated that the expression of fatty acid-binding protein (FABPL_RAT) in the liver was enhanced in the PBPi-treated animals in a dose-dependent manner while the expression of this protein was suppressed in the untreated STZ animals. This is similar to the findings of Melki and Abumrad [42]. The result suggests that PBPi contributes to the enhanced abundance of FABPs in this study. Taken together, this study provides significant insights into some of the mechanisms underlying the efficacy of PBPi treatment in the management of diabetes in STZ-diabetic animal models.

## 4. Materials and Methods

### 4.1. Chemicals

All chemicals and reagents used this study were procured from Sigma Chemical Co. (St. Louis, MO, USA) and Merck (Modderfontein, South Africa); they are of analytical grade or equivalent.

### 4.2. Plant Material and Extract Preparation 

Fermented *Parkia biglobosa* seeds were obtained from a local market in Ijebu-Ode, Ogun State, Nigeria. An import permit (P0060156) from the Department of Agriculture, Forestry and Fisheries (DAFF; Pretoria, South Africa) was obtained. The seed sample was authenticated in the Department of Botany, University of Zululand; and a voucher specimen (B07) was deposited in University Herbarium.

The fermented seeds of *Parkia biglobosa* were oven-dried (50 °C) and pulverized using an electric blender to obtain a coarse powder. One kilogram of uniform powdered seeds was defatted with hexane to obtain the defatted extract. The defatted extract was air-dried and then extracted (1:10 *w*/*v*) with butanol to remove possible anti-nutrients. Protein isolate was obtained from the defatted extract using the method described by Nkosi and co-workers [43]. Briefly, the dry defatted extract was re-suspended in distilled water at pH 10. Thereafter, the resultant suspension was filtered to remove debris and the filtrate adjusted to pH 5, followed by centrifugation at 7650× *g* for 15 min at 4 °C. The supernatant was discarded while the pellet containing the protein isolate was retained and freeze-dried to yield a brown extract. The lyophilized extract was kept dry until needed. 

### 4.3. Experimental Animals and Experimental Design

Sprague Dawley rats (weighing about 250–290 g) used in this study were obtained from the animal house of the Department of Biochemistry and Microbiology, University of Zululand. The animals were kept for acclimatization for seven days prior to the commencement of the study; they were maintained at standard conditions of temperature and relative humidity, with a 12 h light/dark cycle. The animals were provided with standard rat pellets and water ad libitum. The animal experimental protocols were in accordance with the recommendations of the University of Zululand Institutional Animal Ethical Committee (UZREC 171110-030-RA level 02 Dept 2014/74).

### 4.4. Induction of Diabetes and Experimental Design

Following an overnight fasting, diabetes was induced in the selected rats by a single intraperitoneal injection of freshly prepared STZ (Sigma-Aldrich Co., St. Louis, MO, USA) at a dose of 60 mg/kg body weight; dissolved in 0.1 M ice cold citrate buffer, pH 4.5 [44]. Diabetes was confirmed in the rats 72 h after STZ administration by measuring their fasting blood glucose (FBG) levels. Rats with FBG level above 300 mg/dL were considered diabetic and selected for the study. Results of the changes in blood sugar by PBPi is not reported in this study because it has been reported in a separate manuscript that is currently under review in another journal. Treatment commenced on the fourth day and continued for a period of 28 days [15]. Forty healthy, male rats (averaging 12 weeks old), were divided into 4 groups of 10 animals each and treated as follows: Group 1 (control) was given citrate buffer only. Group 2 (STZ), diabetic control. Group 3 (STZ PI 200) was diabetic and received protein isolate (200 mg/kg body weight). Group 4 (STZ PI 400) were diabetic rats that received protein isolate (400 mg/kg body weight). Treatments were given orally for 28 days.

### 4.5. Processing of Tissue Samples and Protein Extraction

At the end of the 28 days of treatment, the rats were fasted overnight and then sacrificed under anesthesia. The livers were removed, washed in saline, blotted dry, and weighed. Total protein from the liver of each experimental animal within each group was extracted from approximately 800 mg of the freshly collected liver samples. The liver samples were homogenized on ice with IKA T25 Ultra Turrax high speed homogenizer in ice cold protein extraction buffer comprising of 50 mM Tris-HCl, 1% Triton X-100, 150 mM NaCl, 5 mM EDTA, 0.02% NaN_3_, 100 mM PMSF pH 7.4 and freshly prepared dithiothreitol (DTT) to a final concentration of 10 mM. The homogenized samples for the different treatment groups were pooled together and kept on ice for 30 min and vortexed at intervals [45,46]. Subsequently, the homogenates were centrifuged at 20,000× *g* for 10 min at 4 °C to pellet the debris. The supernatant was immediately collected and the total protein concentration was estimated by measuring absorbance at A_280nm_ with a NanoDrop ND1000 spectrophotometer (Thermo Fisher Scientific, Waltham, MA, USA). Thereafter, the supernatants containing the protein samples were stored at −80 °C until needed for further use. 

### 4.6. 1D SDS-PAGE, In-Gel Digestion and Peptide Extraction 

Twenty micrograms of the liver proteins of diabetic rats and healthy controls were separated on SDS-PAGE gels. Gel bands were destained with 200 μL of 50% acetonitrile/25 mM Ammonium bicarbonate (NH_4_HCO_3_) until clear. Samples were dehydrated and desiccated with 100 μL acetonitrile (ACN) before reduction with 2 mM Tris (2-carboxyethyl) phosphine (TCEP) in 25 mM NH_4_HCO_3_ for 15 min at room temperature with agitation. Excess TCEP were removed and cysteine residues were carbamidomethylated with 20 mM iodoacetamide (Sigma) in 25 mM NH_4_HCO_3_ for 30 min at room temperature in the dark. After carbamidomethylation, the gel pieces were washed with 25 mM NH_4_HCO_3_ followed by another dehydration step. Proteins were digested by rehydrating the gel pieces in trypsin (Promega) solution (10 ng/μL) and thereafter incubated at 37 °C overnight. Peptides were extracted from the gel pieces once with 30% acetonitrile; 0.1% Trifluoroacetic acid (TFA) (Sigma) for 45 min at room temperature with occasional vortexing. The samples were dried down to remove residual NH_4_HCO_3_ and were re-dissolved in 0.1% Trifluoroacetic acid (TFA) and were purified and concentrated using C_18_ ZipTip^®^ (Merck, Darmstadt, Germany) according to manufacturer’s instructions. The purified samples were eluted in 50% acetonitrile/H_2_O containing 0.1% TFA and dried in a speed vac followed by resuspension in 10 μL 0.1% TFA. 

### 4.7. nLC Procedure 

Peptides from the four purified samples (bands in the region of 10–15 kDa: lane 2, lane 4, lane 6, and lane 8) were separated using nLC-MS. It should be noted that for the purpose of this study, peptides from samples on lane 3, lane 5, and lane 7 were not analyzed. All experiments were performed on a Thermo Scientific EASY-nLC II connected to a Proteineer fc II protein spotter controlled through HyStar software. For liquid chromatography, separation was performed on an EASY column (2 cm, 75 μm ID, 5 μm, C_18_) pre-column followed by an analytical column (10 cm, 75 μm ID, 3 μm, C_18_) with a flow rate of 100 μL/h using a 48 min gradient run.

### 4.8. Mass Spectrometry 

MALDI-TOF MS and LIFT MS/MS were performed using an UltrafleXtreme MALDI ToF/ToF system (Bruker Daltonics, Bremen, Germany) with instrument control through Flex control 3.4. Peptides were ionized with a 337 nm laser and spectra acquired in reflector positive mode at 28 kV using 100 laser shots per spectrum with a scan range of *m*/*z* = 700–4000. Spectra were internally calibrated using peptide calibration standard II (Bruker Daltonics, Bremen, Germany). Peptide spectra of accumulated 4000 shots were automatically processed using WARP LC 3.2 software (Bruker Daltonics, Bremen, Germany).

### 4.9. Data Analysis

Database interrogation was performed with the Mascot algorithm using the Swiss-Prot database on a ProteinScape 3.0 workstation (Bruker Daltonics Inc., Billerica, MA, USA). The search parameters were as follows: Taxonomy—*Rattus rattus*, Enzyme—trypsin; Missed cleavages—1; Fixed modification—carbamidomethyl (C); Variable modification—Oxidation (M) and Deamidation (NQ); Precursor tolerance—50 ppm; Fragment tolerance—0.7 Da. Candidate protein matches with molecular weight search (MOWSE) score greater than 22 were considered as identified proteins.

## Figures and Tables

**Table 1:** Summary of all differentially expressed proteins identified form rat liver by nLC-MS.

S/N	Accession No.	Protein Name	Observed MW(kDa)/pI
1	THIO_RAT	Thioredoxin OS = Rattus norvegicus GN = Txn PE = 1 SV = 2	11.70/4.64
2	PROF1_RAT	Profilin-1 OS = Rattus norvegicus GN = Pfn1 PE = 1 SV = 2	14.90/9.43
3	MPC1_RAT	Mitochondrial pyruvate carrier 1 OS = Rattus norvegicus GN = Mpc1 PE = 2 SV=1	12.40/10.40
4	FABPL_RAT	Fatty acid-binding protein, liver OS = Rattus norvegicus GN = Fabp1 PE = 1 SV = 1	14.30/8.96

**Table 2:** Differentially expressed proteins identified from rat liver in each experimental group by nLC-MS.

S/N	Accession No.	Control		STZ		STZ PI 200		STZ PI 400	
		MOWSE Score	No. of Peptides/Seq. Coverage	MOWSE Score	No. of Peptides/Seq. Coverage	MOWSE Score	No. of Peptides/Seq. Coverage	MOWSE Score	No. of Peptides/Seq. Coverage
1	THIO_RAT	65.90	1/8.60 (%)	-	_	-	-	-	-
2	PROF1_RAT	24.62	1/7.90 (%)	266.82	6/36.70 (%)	-	-	-	-
3	MPC1_RAT	-	-	-	-	-	-	23.47	1/7.30 (%)
4	FABPL_RAT	275.54	7/33.10 (%)	23.51	1/4.80 (%)	68.58	1/14.20 (%)	126.67	3/31.50 (%)
